# Electrospun Hyaluronan Nanofiber Membrane Immobilizing Aromatic Doxorubicin as Therapeutic and Regenerative Biomaterial

**DOI:** 10.3390/ijms24087023

**Published:** 2023-04-10

**Authors:** Xiaowen Han, Mingda Zhao, Ruiling Xu, Yaping Zou, Yuxiang Wang, Jie Liang, Qing Jiang, Yong Sun, Yujiang Fan, Xingdong Zhang

**Affiliations:** 1 National Engineering Research Center for Biomaterials, Sichuan University, Chengdu 610064, China; 2 College of Biomedical Engineering, Sichuan University, 29 Wangjiang Road, Chengdu 610064, China; 3 Sichuan Testing Center for Biomaterials and Medical Devices, Sichuan University, 29 Wangjiang Road, Chengdu 610064, China

**Keywords:** electrospinning, drug delivery, postoperative therapy

## Abstract

Lesioned tissue requires synchronous control of disease and regeneration progression after surgery. It is necessary to develop therapeutic and regenerative scaffolds. Here, hyaluronic acid (HA) was esterified with benzyl groups to prepare hyaluronic acid derivative (HA-Bn) nanofibers via electrospinning. Electrospun membranes with average fiber diameters of 407.64 ± 124.8 nm (H400), 642.3 ± 228.76 nm (H600), and 841.09 ± 236.86 nm (H800) were obtained by adjusting the spinning parameters. These fibrous membranes had good biocompatibility, among which the H400 group could promote the proliferation and spread of L929 cells. Using the postoperative treatment of malignant skin melanoma as an example, the anticancer drug doxorubicin (DOX) was encapsulated in nanofibers via hybrid electrospinning. The UV spectroscopy of DOX-loaded nanofibers (HA-DOX) revealed that DOX was successfully encapsulated, and there was a π–π interaction between aromatic DOX and HA-Bn. The drug release profile confirmed the sustained release of about 90%, achieved within 7 days. In vitro cell experiments proved that the HA-DOX nanofiber had a considerable inhibitory effect on B16F10 cells. Therefore, the HA-Bn electrospun membrane could facilitate the potential regeneration of injured skin tissues and be incorporated with drugs to achieve therapeutic effects, offering a powerful approach to developing therapeutic and regenerative biomaterial.

## 1. Introduction

The limited self-repair capability of diseased tissues led to the development of bifunctional scaffolds, which provide topographical biomimetic and mechanical support to regulate cell morphology, proliferation, and tissue regeneration [1,2,3,4]. Concomitantly, it also requires scaffolds to deliver a broad range of drugs, and other bioactive substances, in a controlled manner to prevent infection and improve the efficacy of treatment [5,6,7]. Electrospinning is a versatile and efficient technique for generating fibers in the sub-micrometer range, giving rise to nanofibrous scaffolds with favorable surface chemical properties for drug incorporation. In addition, the high surface-to-volume ratios and porosity make them beneficial to improving drug loading efficiency as well as mass transfer properties [8]. The high similarity between electrospun membranes and native extracellular matrix (ECM) facilitates cell adhesion and growth, while inherently guiding cellular drug uptake [9,10,11,12,13]. Moreover, nanofibers can be further functionalized via incorporation with bioactive factors and/or functional groups to boost their regenerative or therapeutic performance [14,15,16]. These advantages enable bifunctional electrospun fiber scaffolds to mimic the functions of living systems, thereby expanding the application of electrospun fibers in the fields of tissue regeneration and disease treatment. Remarkable progress has been made regarding the development of electrospinning methods to regulate their structures and properties, including fiber diameter, alignment, and pattering to control cell proliferation, migration, and differentiation for enhancing the repair or regeneration of various types of tissues [17]. Thus far, the principle of electrospinning has been well documented in the literature. Additionally, great progress on the modification of the instrument has been made to increase the capability and versatility of electrospinning. The processing flexibility of electrospinning warrant nanofiber generation from various types of precursor materials, such as naturally derived biopolymers, synthetic polymers, small molecules, colloidal particles, or their combinations. A variety of biocompatible polymers including collagen, HA, dextran, chitosan, poly (lactic acid) (PLA), and poly(ε-caprolactone) (PCL), have been utilized to eletcrospin fibrous scaffolds for tissue engineering and therapeutic applications [18].

Hyaluronic acid (HA), as a component of the ECM, is biocompatible and biodegradable. It plays essential roles in inflammation, angiogenesis, and immune surveillance, thereby participating in tissue homeostasis and tissue repair [19,20,21,22,23]. The CD44 receptor on the endothelial cell surface interacts with HA, triggering cell migration and sprouting, resulting in angiogenesis [24]. HA with a high molecular weight (over 1 × 10^6^ Da) presents anti-inflammatory properties by modulating inflammatory cell migration and activation and protecting parenchymal cells from leukocyte-mediated death [25]. Hydroxyl and carboxyl groups on its backbone endow the polysaccharide with a high degree of hygroscopicity, enabling exudate absorption and hydration. When under physiological conditions, the HA swells to form a hydrated network of endued tissues with plasticity that is essential to tissue development and remodeling. Unlike collagen extracted from animals, which has large batch-to-batch variance and immunogenicity. HA can be produced by microbial fermentation with increased stability avoiding immunogenicity issues [26]. Moreover, the reactive groups of HA permit the chemical functionalization of the backbone, which is useful for obtaining customized derivatives [27,28]. These advantages make HA ideal for various biomedical applications, such as drug delivery [29], wound healing [30], cartilage repair [31], nerve regeneration [32], etc. Although the hydrophilicity of HA allows it to absorb exudate, which is beneficial for wound repair and joint lubrication; however, it is also detrimental to cell adhesion and subsequent tissue formation [33,34]. Hydrophobic modification (such as esterification) of HA can improve its cell adhesion ability and promote cell spreading. Additionally, HA is not electrospinnable in its natural condition. It is often blended with other polymers or modified to improve its spinnability [35,36]. Although, in the literature, there have been discussions on the electrospinning of HA derivatives such as HAMA [37] to prepare nanofibers, the electrospinning of their esterified derivatives (HA-Bn) has yet to be reported. More to the point, the benzyl group of HA-Bn may facilitate hydrophobic drugs containing benzene rings binding to HA-Bn nanofibers through π–π interaction to achieve effective drug loading and sustained release. Doxorubicin (DOX) is a widely employed anticancer drug containing a benzene ring. It is also used as an adjuvant chemotherapy drug for the postoperative treatment of melanoma. When DOX is loaded into the surface of nanofibers, an undesirable burst release is inevitable, causing severe side effects [38,39]. We believed this issue could be addressed with the aid of the π–π interaction between DOX and HA-Bn.

Giving credence to this, we prepared esterified hyaluronic acid (HA-Bn) nanofibers, via electrospinning, and explored their biocompatibility. HA-Bn nanofibers greatly promoted cell spreading and proliferation in vitro. Moreover, HA-Bn nanofibers could be loaded with DOX to produce a fibrous membrane with sustained drug release and anticancer activity in vitro.

## 2. Results and Discussion

### 2.1. Characterization of HA-Bn Nanofibers

**Figure 1:**
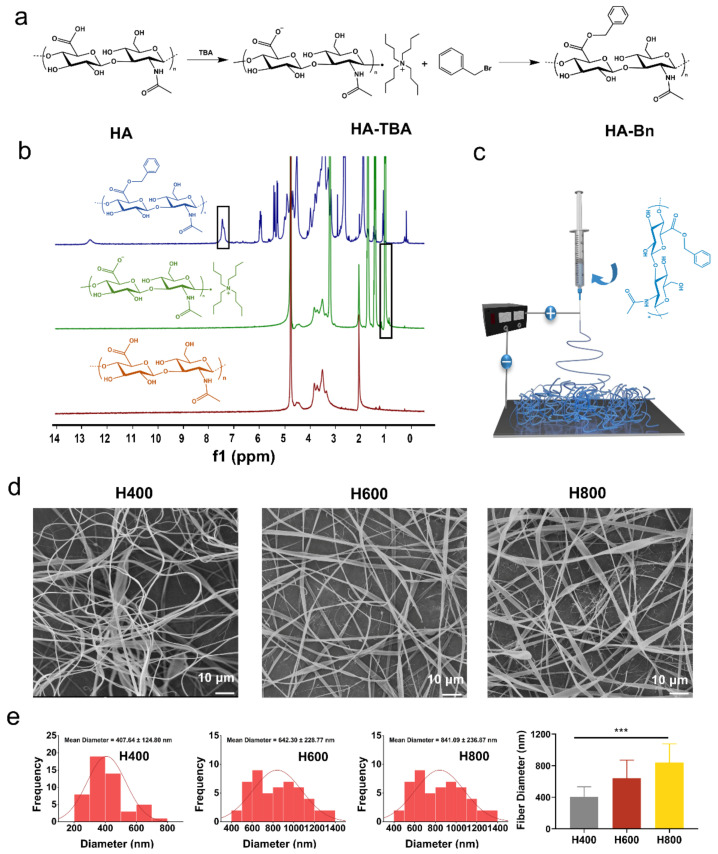
(**a**) Synthetic route of HA-Bn. (**b**) ^1^H-NMR of products. (**c**) Schematic diagram of HA-Bn electro spinning. (**d**) SEM images and (**e**) corresponding diameter distributions of different diameter nanofibers. (*** indicates *p* < 0.001).

The molecular structures of products in each procedure were analyzed via ^1^H-NMR (Figure 1b). The NMR spectrum exhibited the characteristic resonance signals of methyl in Hyaluronic acid tetrabutylammonium (HA-TBA) at 1 ppm and the sugar ring of HA from 3 to 4 ppm, confirming the successful synthesis of HA-TBA [40]. The characteristic peak of benzyl groups at 7.5 ppm proved that the benzyl ester had been successfully grafted onto HA. The degree of esterification was calculated from the peaks of the spectrum at 13 ppm for the carboxyl group residues of HA and the peaks at 7.5 ppm for the benzyl groups, namely 72%.

It was found that human fibroblasts had a well-spread morphology as well as upregulated collagen gene expression on membranes with a fiber diameter of 350–1100 nm [41]. Additionally, dermal fibroblast proliferated better on membranes with small diameter fibers than membranes composed of fibers with a diameter of around 1 μm [41,42]. HA-Bn electrospun membranes with varying fiber diameters of about 400 nm, 600 nm, and 800 nm were prepared and named as H400, H600, and H800, respectively, by adjusting the process parameters (Table 1). The effect of electrospinning parameters on fiber size and morphology have been well investigated and are universally acknowledged. Applied voltage, feeding rate, and collecting distance are the major factors controlling fiber sizes [17,43]. In general, higher voltage, larger distance between the tips and collector, and a slower flow rate are all combining factors which produce thinner fibers due to the higher electrostatic repulsive forces against surface tension, as well as better stretching and elongation of the jet, and vice versa [44,45,46]. In this context, the mean diameter of electrospun fibers can be easily tuned by adjusting these parameters. The structure of the fibrous membrane and the corresponding fiber diameter distribution were characterized by SEM and image J. It can be seen from Figure 1d that the morphology of the three groups of fibrous membranes were all continuous nanofibers without obvious beading, which presented a random orientation. The diameter distributions were 407.64 ± 124.8 nm (H400), 642.3 ± 228.76 nm (H600), and 841.09 ± 236.86 nm (H800). Fiber diameter differences were statistically significant (*p* < 0.001) (Figure 1e).

### 2.2. The Biocompatibility of HA-Bn Nanofibers In Vitro

**Figure 2:**
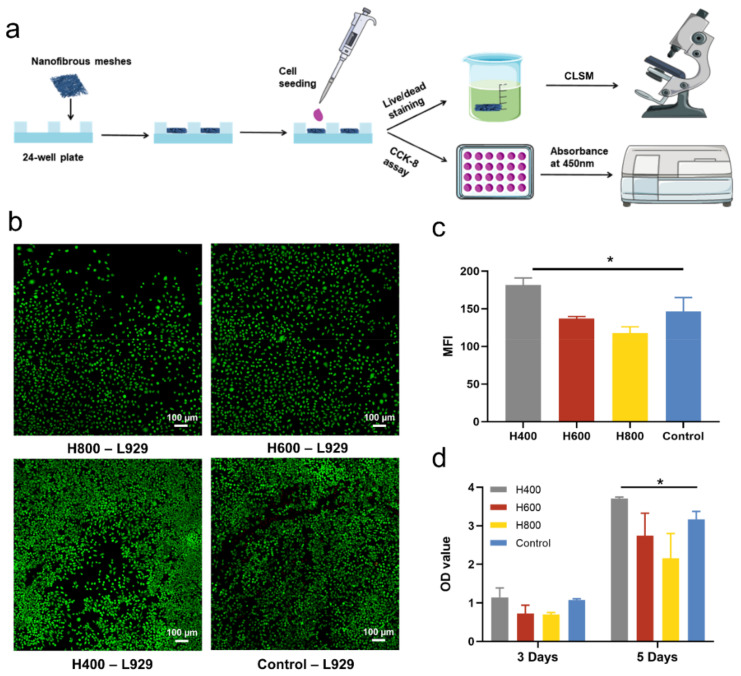
(**a**) Schematic diagram of cell viability test. (**b**) Live/dead staining of L929 cells cultured on nanofibers for 3 days and (**c**) corresponding mean fluorescence intensity of FDA staining (n = 3). (**d**) CCK-8 assay of L929 cells on nanofibers (* indicates *p* < 0.05).

In order to assess the effects of different diameters of electrospun fiber membranes on cell proliferation and growth, we conducted in vitro cell experiments, and the experimental processes are summarized in Figure 2a. Fibroblast L929 were seeded and cultured on electrospun fiber membranes with different diameters. The cell viability and proliferation were evaluated via live/dead staining and the cell counting kit-8 assay. As shown in Figure 2b, after being cocultured with HA-Bn membranes for 3 days, the majority of the L929 cells were stained in green, and dead cells (red) could be neglected, indicating a good cytocompatibility of the membranes. Moreover, cells of all membrane groups were rounded rather than elongated on fibers. As described in the literature, although cells generally elongate along with the fiber alignment direction on oriented aligned fibers, they appear round and satellite morphology on the randomly oriented nanofibers while elongating on randomly oriented microfibers. This may contribute to the fact that the focal adhesions of cells were larger but fewer on microfibers compared to nanofibers [47]. The quantified mean fluorescence intensity of live cells in four groups revealed that, after 3 days of culture, the cell density on the H400 membranes was significantly higher than that of other groups (*p* < 0.05, Figure 2c). This result was also supported by the CCK-8 assay. Figure 2d showed that the OD value of H400 and the control groups were significantly higher than that of the other two groups on day 3. After incubation for 5 days, cells on H400 membranes proliferated the most, even surpassing the control group. This was consistent with our previous research, which revealed that fiber diameter could influence cell proliferation and viability. It has been reported that cells proliferate more on membranes with smaller fiber diameters. The elevated expression of extracellular matrix-related genes, such as collagen I, III and IV, and cell proliferation makers were observed with decreasing fiber diameter, which led to the increase of cell viability [48,49]. Overall, fiber diameter may have varying degrees of effects on cell proliferation and morphology on a cell-type dependent manner. Our results indicated that the fibroblast could proliferate continually on the HA-Bn membranes, and the H400 membrane was more conducive for the proliferation of fibroblast in vitro.

### 2.3. Cell Morphology on HA-Bn Nanofibers

**Figure 3:**
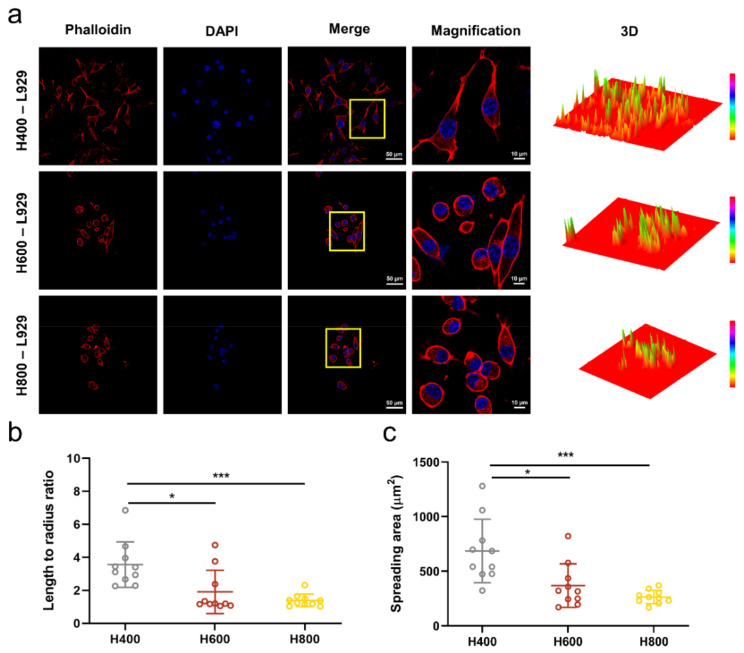
(**a**) Phalloidin/DAPI staining of L929 cells seeded onto nanofibers after 3 days and 3D reconstruction of red fluorescence by image J. (**b**) Quantification of cell length to radius ratio and (**c**) spreading area of L929 cells (n = 10). The yellow boxes indicate the magnification field of view (*** indicates *p* < 0.001, * indicates *p* < 0.05).

The cytoskeleton staining of cultured L929 cells on the fibrous membranes was performed to visualize the cell spreading more intuitively. The fluorescence images of the cytoskeleton (red) and nucleus (blue) in Figure 3a showed that, as the diameter of fibers decreased, more cells adhered to the electrospun membrane in the H400 group, and were distributed in a spindle-like shape, demonstrating better cell attachment and spreading than other groups. The semi-quantitative analysis was consistent with this result. The length-to-radius ratio and cell-spreading area of cells on H400 membranes were significantly higher than those on the H600 and H800 membranes (Figure 3b,c). These results indicated that H400 membranes could significantly promote cell adhesion and spreading in vitro, which is beneficial to tissue regeneration.

### 2.4. Characterization of HA-DOX Nanofibers

**Figure 4:**
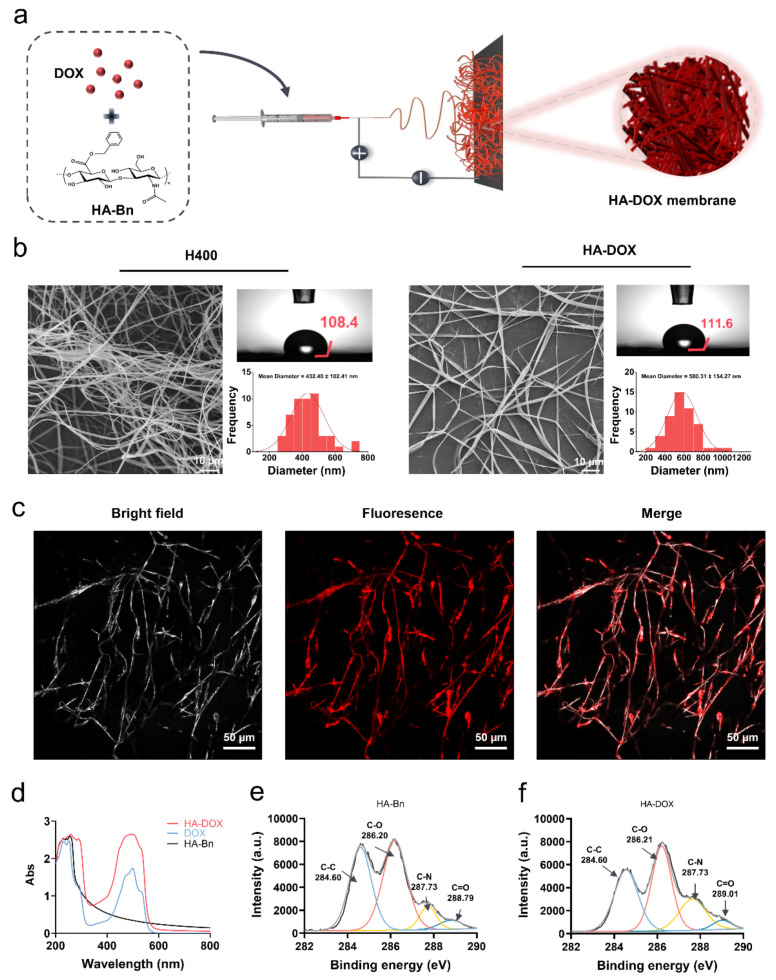
(**a**) Schematic diagram of HA-DOX electrospinning. (**b**) SEM and water contact angle images of nanofiber with or without DOX, and corresponding diameter distribution. (**c**) CLSM images of HA-DOX nanofiber. (**d**) Ultraviolet absorption spectrum of samples. (**e**) C 1s XPS spectrum of HA-Bn and (**f**) HA-DOX.

Hybrid electrospinning is the most straightforward technique used to deposit a nanofibrous matrix loaded with drugs. Since the distribution of drugs is random, the release profile usually manifests as an instant or burst release followed by a moderate release [50,51]. As a result of the accumulation of drugs and the high specific surface area of nanofibers, drugs were usually fully released from fibers within a short timescale, which is incompatible with long-term drug administration demand. In our design, the benzyl group of HA-Bn may facilitate hydrophobic drugs containing benzene rings binding to HA-Bn nanofibers through π–π interaction, thereby avoiding the explosive release of drugs and realizing sustained drug release. To verify our hypothesis, the anticancer drug DOX was added to the HA-Bn electrospinning solution to prepare drug-loaded HA-DOX nanofibers for electrospinning by using the same electrospinning parameter as that of the H400 membranes (Figure 4a). SEM and water contact angle tests were performed to characterize the HA-DOX membrane. It could be seen that the average fiber diameter increased from 432.40 ± 102.41 nm to 580.31 ± 154.27 nm after being loaded with DOX, while there was no significant change in the water contact angle (Figure 4b). DOX has an emission wavelength of 615 nm, the DOX loading of the HA-DOX nanofiber was observed by CLSM [52]. It can be seen from Figure 4c that DOX was evenly distributed with HA-DOX nanofibers, confirming the effective loading of drugs. UV absorption spectrum analysis and X-ray photoelectron spectroscopy (XPS) were carried out to explore whether there was π–π interaction between HA-Bn and DOX. As presented in Figure 4d, all groups showed the UV absorption spectra at 230–270 nm, which was assigned to the π–π* transition of benzene rings [53,54]. DOX exhibited an absorption peak at 490 nm, while HA-Bn had no absorption in this range [55]. After being loaded with DOX, the absorption intensity of HA-DOX remarkably strengthened, even compared with DOX at the same concentration. This phenomenon could be attributed to the π–π interaction between HA-Bn and DOX [53,56]. In Figure 4e,f, the XPS C 1s spectra also confirmed that the binding energy of C=O shifted from 288.79 eV in HA-Bn to 289.01 eV in HA-DOX. This was ascribed to the fact that the C=O chemical bonding in HA-DOX gained fewer electrons from the aromatic rings due to the π–π interaction, consequently endowing these one-dimensional molecular tunnels with higher reaction activity [57]. Although the surface functionalization of electrospun fibers has been studied extensively, water-soluble natural polymers were often considered to be difficult to directly process into nanofibers due to factors related to their unstable nature, such as being vulnerable to processing conditions [58]. Therefore, most of the functional nanofibers were synthetic polymers. The hydrophobic modification method adopted in this work not only improved the spinnability of HA and avoided the use of toxic chemical reagents required for HA crosslinking but also enabled the encapsulation and sustained release of the anticancer drug DOX through the π–π interaction between aromatic DOX and HA-Bn, expanding the versatility of functionalized nanofibers.

### 2.5. Drug Release and In Vitro Degradation

**Figure 5:**
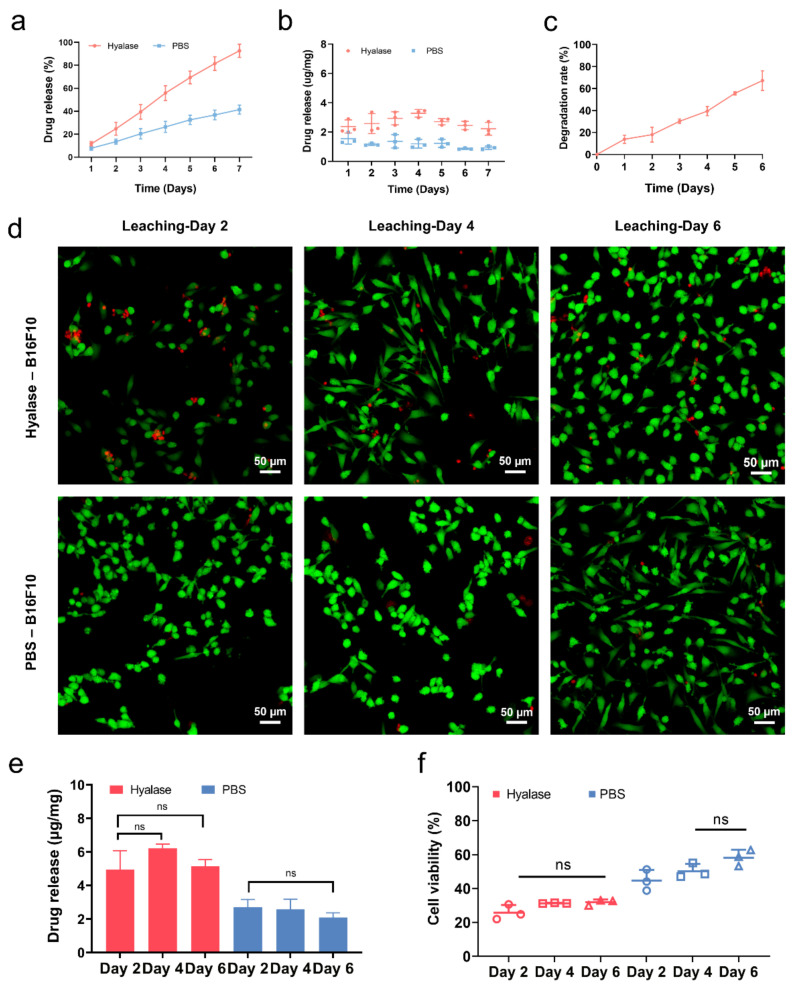
(**a**) The cumulative drug release profile of HA-DOX in PBS/Hyalase. (**b**) The drug release amount of DOX per day. (**c**) In vitro degradation. (**d**) Live/dead staining of B16F10 cells cultured with every two days of leaching. (**e**) Drug release profile of HA-DOX for every two days. (**f**) CCK-8 assays of B16F10 cells cultured with every two days of leaching (ns indicates no significance).

The drug release profile of HA-DOX nanofibers was assessed by soaking the membranes in saline/Hyaluronidase solution and evaluating the release of DOX through the UV spectrum. The cumulative drug release is presented in Figure 5a. In the absence of an enzyme, DOX was slowly released from the membranes with negligible initial burst release. Under the catalysis of hyaluronidase, DOX release increased with fiber degradation and reached 92.7% after 7 d. Figure 5b plots the drug release amount of DOX per day. Sustained and uniform drug release was observed in both PBS and Hyaluronidase solution, indicating that DOX was not passively absorbed but bound to nanofibers through π–π interaction. The in vitro degradation of nanofibers is shown in Figure 5c. The nanofibers slowly degraded under the enzymatic hydrolysis, and the degradation rate reached 68.74% after 6 d. This result further demonstrated that DOX was gradually released as the nanofibers degraded, rather than by diffusion. Failure to prevent a burst release may result in undesired toxicity associated with high concentrations of drugs. The sustained drug release of HA-DOX nanofibers proved that binding to the HA-Bn through π–π interaction may thus be a feasible approach to stabilize drugs and could be used for disease treatment.

### 2.6. Anti-Tumor Ability of HA-DOX Nanofibers In Vitro

The anti-tumor ability of the drug-loaded HA-DOX fibrous membranes in vitro was tested against B16F10 cell lines. Live/dead staining graphs (Figure 5a) revealed that the living cells in the Hyalase group were sparse, accompanied by several dead cells, compared to the PBS group. There was no significant difference in the number of living cells after they were cultured with the leaching at three time points of Day 2, Day 4, and Day 6, which was also consistent with the result of the DOX release profile for every 2 days (Figure 5b). The CCK-8 assay showed that the cell viability of Hyalase group was lower than that of PBS group. There was no significant difference in cell viability at the three time points of Day 2, Day 4, and Day 6. For the PBS group, the cell viability of the Day 2 group was slightly lower than that of the Day 4 and Day 6 groups. The cell viability of most groups was under 50%, suggesting that the HA-DOX membrane has good anti-tumor activity in vitro (Figure 5c).

## 3. Materials and Methods

### 3.1. Materials

Sodium Hyaluronate (MW = 100 kDa) was provided by Shandong Freda Biochem Co., Ltd. (Shandong, China). Dowex^®^ 50WX8-200, tetrabutylammonium hydroxide (TBA-OH), benzyl bromide, TritonX-100 and hyaluronidase were purchased from Sigma-Aldrich (St. Louis, MO, USA). Anhydrous dimethyl sulfoxide (DMSO), ethyl acetate, and Hexafluoroisopropanol (HFIP) were obtained from Aladdin Reagent Company (Shanghai, China). Doxorubicin hydrochloride (Dox•HCl, >99%) and cell counting kit-8 (CCK-8) were brought from Dalian Meilun Biology Technology Co., Ltd. (Dalian, China). Fluorescein diacetate (FDA), propidium iodide (PI), 4′,6-diamidino-2-phenylindole (DAPI), and TRITC-phalloidin were purchased from Beijing Solarbio Science & Technology Co., Ltd. (Beijing, China). DMEM medium, RPMI-1640 and fetal bovine serum were provided by Gibco (Cleveland, TN, USA). Penicillin-streptomycin and trypsin were purchased from HyClone (Logan, UT, USA). All other chemicals were used without further purification.

### 3.2. Synthesis and Characterization of HA-Bn

HA-Bn was synthesized according to a previous report with some modifications [59]. A total of two steps were included: Even HA was first converted to its tetrabutylammonium salt (HA-TBA) and then reacted with benzyl bromide. The synthetic route is shown in Figure 1a. First, 1 g sodium salt of HA was dissolved in 100 mL of DI water, and polymeric ion exchange resin Dowex^®^ 50WX8-200 (3 g) was conditioned prior to use to remove residuals before being added to HA solution and mixed for 4 h. After mixing, the Dowex resin was collected and removed by centrifugation at 4000 rpm for 5 min. Then, 1.4 M of excess TBA-OH was added to the supernatant and mixed for 30 min. The HA-TBA solution was then transferred, frozen, and lyophilized for 3 d.

A total of 1 g of HA-TBA was then solubilized in 100 mL of DMSO at room temperature before adding 0.55 g of benzyl bromide. The mixture was allowed to react at 30 °C for 12 h. The resulting mixture was added dropwise to 500 mL of ethyl acetate under vigorous agitation. A precipitate was formed, which was collected and washed three times with 500 mL of ethyl acetate and, finally, vacuum dried for at least 48 h. Products were characterized by ^1^H-nuclear magnetic resonance spectra (^1^H-NMR, 400 MHz, Bruker AMX-400, Chicago, IL, USA).

### 3.3. Fabrication of HA-Bn and HA-DOX Nanofibers

To fabricate the HA-Bn electrospun membrane with different fiber diameters, HA-Bn with an optimized concentration (7 wt%) was dissolved in hexafluoroisopropanol (HFIP) by stirring in order to prepare an electrospinning solution. Subsequently, the solution was loaded into a 2.5 mL syringe connected to a 20 G needle with blunt tips. The needle-collector distance was adjusted to 9 cm, and the flow rate was fixed at 1 mL/h to obtain continuous flow. The voltage power supply was set at 21 kV to generate nanofibers with a mean diameter of around 400 nm (H400). The process parameters were changed to prepare nanofibers with other diameter distributions. The schematic diagram of HA-Bn electrospinning is shown in Figure 1c, and the electrospinning parameters for different HA-Bn membranes are summarized in Table 1.

To fabricate HA-DOX nanofibers, DOX was added to the HA-Bn electrospinning solution at a concentration of 2 mg/mL. This drug-loaded solution was then fed to the aforementioned electrospinning setup to fabricate HA-DOX nanofibers. The schematic diagram of HA-DOX electrospinning is shown in Figure 4a. All electrospinning processes were performed at ambient temperature, and the prepared nanofibers were carefully collected and dried for 48 h to remove reagent residues.

### 3.4. Characterization of Electrospun Membranes

Scanning electron microscopy (SEM): The morphologies of the electrospun membranes (H400, H600, H800, HA-DOX) were observed via scanning electron microscopy (SEM, HITACHI S-4800). Before observation, the membranes were sputter-coated with gold. The mean fiber diameter and diameter distribution were determined by Image J based on the SEM images.

Water contact angle: The wettability of electrospun membranes was assessed via water contact angle measurements. Water drops of 8 µL were generated by a micropipette and carefully deposited on the surface of the specimens. Photographs of the droplets on the surface were acquired using a digital camera, and the contact angles were measured.

The CLSM (LSM 880, Zeiss, Jena, Germany) was applied to observe the DOX loading in HA-DOX nanofibers. DOX could be observed at an emission wavelength of 615 nm.

Additionally, X-ray photoelectron spectroscopy (XPS): The XPS (Kratos AXIS ULTRA DLD, Manchester, UK) was proceeded to explore the interaction between HA-Bn and DOX. A binding energy value of C1s (284.6 eV) was selected as the standard for energy correction.

### 3.5. Drug Release Profile and In Vitro Degradation

The drug release of HA-DOX in vitro was tested via dialysis in PBS and hyalase (0.4 mg/mL) solution. Briefly, a HA-DOX (containing 20 ug/mg of DOX) mat was cut into small samples (~5 mg) and accurately weighed. Each sample was then immersed in 2 mL of PBS or hyalase medium and incubated at 37 °C with shaking at 60 rpm. At each time interval, 600 µL release medium was collected for measurement, and 600 µL fresh medium was added. The DOX in the release medium was measured using a multifunctional microporous plate detector (H1M, BioTek Instruments, Winooski, VT, USA) in a 96-well plate at 490 nm. Drug release was determined as the percentage ratio of the mass of the released DOX at each time interval, normalized by the mass of DOX in the original sample. All of the steps were protected from light.

The in vitro degradation was assessed using the same enzymatic degradation media of drug release test. The samples were accurately weighed and immersed in 2 mL of enzymatic solution before being incubated at 37 °C for 6 consecutive days. At each time inerval, the samples were collected and lyophilized. The samples were weighted and the degradation rate was calculated.

### 3.6. Cell Viability and CELL Morphology

Mouse fibroblast cells L929 were chosen to evaluate the compatibility of HA-Bn nanofibers. L929 cells (Cell Bank of the Chinese Academy of Sciences) were cultured in DMEM medium with 10% (*v*/*v*) fetal bovine serum (FBS), and 1% penicillin-streptomycin at 37 °C and 5% CO_2_ humidified atmosphere. H400, H600, and H800 membranes were sterilized and placed in 24-well plates and then seeded with L929 cells at a density of 5 × 10^4^ cells per well. After incubation for 3 days, the culture medium was carefully removed and samples were dyed with FDA/PI solution for live/dead assay, and DAPI/ phalloidin for cytoskeleton staining and then observed by CLSM (LSM 880, Zeiss). For live/dead assay, the FDA and PI staining solution were diluted with PBS at a concentration of 5 μg/mL and 1 μg/mL, respectively, before application. The fibrous membranes seeded with cells were washed twice with PBS solution and then stained with FDA/PI staining solution for 3 min. For cytoskeleton staining, membranes with cells were fixed with 4% paraformaldehyde for 30 min, then washed three times with PBS solution. Subsequently, membranes were submerged in 0.1% TritonX-100 for 10 min to improve the permeability of the cell membrane before being stained with 100 μM phalloidin staining solution overnight at 4 °C. Before CLSM observation, the membranes were taken out, washed with PBS solution for 3 min, and stained with 100 μM DAPI staining solution. All staining procedures were performed in the dark.

Cell proliferation was further evaluated using the CCK-8 assay following the manufacturer’s instruction at 3 and 5 days. Namely, the culture medium in the 24-well plate was carefully aspirated and the plate was washed twice with PBS solution. The CCK-8 stock solution was diluted ten times with serum-free DMEM medium before being added to the well plate at a concentration of 1 mL/well. The plate was then placed in an incubator for 2 h in the dark, and finally, 200 μL of the reaction solution was pipetted into a 96-well plate, and the absorbance was detected as the wavelength of 450 nm with a microplate reader. The absorbance of three paralleled wells was measured and recorded.

### 3.7. Statistical Analysis

All data were presented as mean ± standard deviation (SD) of at least three representative experiments. SPSS 22.0 software was used for statistical analysis using one-way ANOVA. The difference was statistically significant at three levels. * *p* < 0.05, ** *p* < 0.01, and *** *p* < 0.001.

## 4. Conclusions

In this study, esterified hyaluronic acid (HA-Bn) nanofiber membranes with different mean fiber diameters were prepared via electrospinning and characterized. HA-Bn membranes showed good biocompatibility. Compared to membranes with larger fiber sizes, the H400 membrane could promote the spreading and proliferation of cells in vitro. Moreover, the anticancer drug DOX was encapsulated in HA-Bn nanofibers via simple blending electrospinning to fabricate HA-DOX membrane. Thanks to the π–π interaction between the benzene rings in DOX and HA-Bn, this drug-loaded membrane could achieve sustained DOX release and have a stable inhibitory effect on B16F10 cells. Therefore, HA-Bn fibrous membranes could not only be used as a simple scaffolds to support potential tissue regeneration, but could also aid aromatic drug loading to achieve controlled release, thereby demonstrating their potential use in the postoperative treatment of diseased tissue.

## Figures and Tables

**Table 1:** Electrospinning parameters of HA-Bn membranes.

Membrane	Flow Rate (mL/h)	Voltage (kV)	Distance (cm)
H400	1	21	9
H600	1	18	9
H800	2	21	9

## Data Availability

Data are contained within the article.
